# The health impacts of preventive cardiovascular medication reduction on older populations: protocol for a systematic review and meta-analysis

**DOI:** 10.1186/s13643-021-01741-2

**Published:** 2021-06-24

**Authors:** Rik S. van der Veen, Joseph J. Lee, Richard J. McManus, Richard F. D. Hobbs, Kamal R. Mahtani, Constantinos Koshiaris, James P. Sheppard

**Affiliations:** Nuffield Department of Primary Care Health Sciences, University of Oxford, Radcliffe Primary Care Building, Radcliffe Observatory Quarter, Woodstock Rd, Oxford, OX2 6GG UK

**Keywords:** Deprescribing, Cardiovascular therapy, Aged, Problematic polypharmacy, Inappropriate prescribing, Multimorbidity, Quality of life, Adverse events, Medication reduction

## Abstract

**Background:**

Polypharmacy is inevitable and appropriate for many conditions, but in some cases, it can be problematic resulting in an increased risk of harm and reduced quality of life. There has been an increasing interest to reduce cardioprotective medications in older adults to potentially reduce the risk of harm due to treatment; however, there is no evidence on safety and efficacy to support this practice currently. This paper describes a protocol for a systematic review on the safety and efficacy of reducing cardioprotective medication in older populations.

**Methods:**

MEDLINE (PubMed), Embase (Ovid), and CENTRAL (Cochrane Central Register of Controlled Trials) will be searched from their inception onwards for relevant studies. Randomised controlled trials and non-randomised studies on interventions (prospective, retrospective cohort, case-control) conducted in older adults (75 years or older) examining reduction of cardioprotective medications will be included. The primary outcome of this study will be all-cause hospitalisation. Secondary outcome variables of interest are all-cause hospitalisation, mortality, quality of life, serious adverse events, major adverse cardiovascular events, falls, fractures, cognitive functioning, bleeding events, renal functioning, medication burden, drug reinstatement, time-in-hospital, and frailty status. Two reviewers will independently screen all citations, full-text articles, and extract data. Confidence in cumulative evidence will be assessed using the GRADE approach; the risk of bias will be assessed by the RoB-II tool for randomised controlled studies and ROBINS-I for non-randomised studies. Where sufficient data are available, we will conduct a random effects meta-analysis by combining the outcomes of the included studies. Sub-group analysis and meta-regression are planned to assess the potential harms and risks of different drug classes and the impacts in different patient populations (e.g. sex, cognitive status, renal status, and age).

**Discussion:**

The study will be a comprehensive review on all published articles identified using our search strategy on the safety and efficacy of cardioprotective medication reduction in the older population. The findings will be crucial to inform clinicians on potential health outcomes of reducing cardiovascular medication in the elderly.

**Systematic review registration:**

PROSPERO CRD42020208223

**Supplementary Information:**

The online version contains supplementary material available at 10.1186/s13643-021-01741-2.

## Background

People are living longer and developing multi-morbidity at old age [1, 2]. As a natural consequence of applying modern treatment guidelines to treat diseases, people are exposed to evermore long-term pharmacotherapeutic interventions [2]. While this increase in pharmacological intervention unquestionably has led to an overall health benefit for patients across disease domains, the surge in interventions has created polypharmacy and problems with inappropriate prescribing in vulnerable populations [3]. The term polypharmacy inadvertently has been associated with negative health impacts, which is why several groups have sought to bring nuance by adopting two variations of polypharmacy; appropriate polypharmacy and problematic polypharmacy [2, 4]. For those patients experiencing problematic polypharmacy and/or inappropriate prescribing there may be an increased risk of adverse events, including falls, hospitalisation, and even mortality [5].

In recent years, there has been an increased interest in medication reduction to address problematic polypharmacy, known as deprescribing. Deprescribing is defined as the process of withdrawal of an inappropriate medication, supervised by a health care professional, with the goal of managing inappropriate polypharmacy and improving outcomes [6, 7]. Medication reduction in older adults is of a particular interest as they are generally not included in large randomised controlled drug efficacy trials and often are multi-morbid and/or frail, which may increase an individual’s risk of adverse events from treatment [8–10].

Cardiovascular drugs have been associated with inappropriate prescribing and their pharmacological action is closely related to negative outcomes seen in problematic polypharmacy-related outcomes, i.e. antihypertensives causing hypotension resulting in syncope and/or falls, and are there for an ideal candidate for withdrawal to potentially improve patient outcomes [5, 8]. Reduction of preventative cardiovascular in drugs in older adults, either prescribed for primary or secondary prevention of cardiovascular disease, can be used to remove potentially inappropriate medications, which could mitigate unwanted risks, like adverse drug reactions [8]. However, preventative cardiovascular medication reduction might increase the risk of cardiovascular events, due to disruption of the normotensive state, increased plaque formation, increased blood coagulation, or increased platelet activity [11–14]. Therefore, the potential benefits and harms of cardiovascular medication reduction need to be balanced against the benefits and harms of continuing therapy, as well as patient wishes.

This study described in this protocol aims to look at the safety and efficacy of cardiovascular medication reduction compared to usual care in patients aged 75 years and over. There are existing systematic reviews that examine the clinical outcomes of reducing cardiovascular drugs, but these provide insufficient evidence to support clinical decision making [15–18]. One systematic review focused on the clinical outcomes of the medication reduction intervention itself, without evidence for the effect of individual drug classes [15]. A systematic review published in 2008 focused on identifying medication withdrawal studies that withdrew a single group or class of drugs, but did not include a meta-analysis due to heterogeneity of included trials [16]. A more focused systematic review looked at antihypertensive medication withdrawal in a broader age group and the proportion of the patients remaining normotensive. Due to the diversity of patients included in this review, no meta-analysis was performed for the clinical outcomes [17]. A recent systematic review reviewed the clinical outcomes of complete antihypertensive withdrawal in the older population, defined as 50 years or older, found no evidence that such an intervention was associated with mortality, heart attack, or stroke, although analyses were almost certainly underpowered [18]. Therefore, this study aims to add to existing evidence by including recently published trials omitted by other publications, inclusion of a wider range of cardiovascular medicines and reduction interventions, and a stricter age selection for patients whom we believe to be at potential increased risk of harm of continued drug exposure [5, 8].

## Methods/design

The present protocol has been registered within the International Prospective Register of Systematic Reviews (PROSPERO) database (registration number CRD42020208223) and is reported in accordance with the Preferred Reporting Items for Systematic review and Meta-Analysis Protocols (PRISMA-P) checklist [19] (Additional File 1).

### Eligibility criteria

Studies will be selected according to the following criteria: study designs, participants, interventions, comparators, outcomes, and setting.

#### Study designs

We will include randomised controlled trials and non-randomised studies of interventions (NRSI). Non-randomised studies of interventions that will be considered are controlled cohort studies (prospective and retrospective) and case-control studies. Studies that are excluded for this study are case reports and studies lacking a comparator.

#### Participants

Studies where at least of half of the population was 75 years of age will be considered for inclusion in this review. Studies including a younger population will be considered if they published a subgroup analysis of the outcomes in patients of 75 years or older. Patients receiving prescriptions either in the context of primary or secondary prevention will be considered for this systematic review. Studies will be excluded if they examine patients receiving palliative care.

#### Intervention

The intervention of interest in this review is deprescribing, where a healthcare professional aims to systematically reduce cardiovascular therapy in older populations to improve outcomes, manage problematic polypharmacy, or prevent adverse events. Preventive cardiovascular therapy is defined as antihypertensives (e.g. angiotensin converting enzyme inhibitors, angiotensin receptor blockers, thiazides, calcium channel blockers, alpha/beta-blockers, central acting antihypertensives), antihyperlipidemics (e.g. statins, fibrates), antiplatelets (e.g. aspirin, P2Y_12_ inhibitors), and anticoagulants (e.g. coumarin-derivatives, direct acting oral anticoagulants).

Excluded are studies in which medication reduction is reactive in nature, e.g. patients experiencing adverse events or symptoms of the drug therapy, and studies that are looking at temporary withdrawal (e.g. in preparation for major surgical interventions) or as part of a run-in phase prior to randomisation in a clinical trial.

#### Comparator

Any study that has a suitable comparator group is considered. Comparisons are considered suitable if they are usual care, placebo-controlled, or active maintenance of current therapy.

#### Setting

Included settings for this review are primary care, nursing homes, and hospital care.

Hospice care setting is excluded from this review.

### Outcomes

#### Primary outcome

The primary outcome of this study will be all-cause hospitalisation regardless of the duration of the stay.

#### Secondary outcomes

Secondary outcomes will include all-cause mortality, quality of life, serious adverse events, major adverse cardiovascular events (defined as nonfatal myocardial infarction, nonfatal stroke, and cardiovascular death), falls (including self-reported and those who need medical attention), fractures, cognitive functioning, bleeding events (major and minor), renal functioning (acute kidney injury and chronic kidney disease), electrolyte anomalies (e.g. hyperkalaemia), change in medication burden (I.e. number of medications prescribed), drug reinstatement, time-in-hospital (length of stay), and change in frailty status.

### Information sources and search strategy

A search strategy using a combination of medical subject headings, publication type, key words, and abstract/title text words has been developed an example for one database (Embase) can be found in Additional File 2. After consultation with an information specialist the databases Embase, MEDLINE, and CENTRAL will be searched from their inception onwards. MEDLINE will be accessed through PubMed, Embase through Ovid, and CENTRAL through the Cochrane Library. The search terms will be adapted for use with each specific database interface. Reference lists of selected articles will be scanned to include additional papers not identified with the search strategy.

#### Language

No restrictions will be placed on the original publication language. A summary table of all articles requiring translation will be made available.

### Study selection

Two review authors (RV and JL) will independently screen the records obtained through the search strategy. Titles and abstracts will be screened against the inclusion/exclusion criteria, before further screening of full-text articles. Differences between the two reviewers will be assessed, reviewed, and resolved by a third reviewer (JS). Study screening, selection, and recording will be done using EndNote (Clarivate Analytics, Philadelphia, USA) and Rayyan [20].

### Data extraction

Two review authors will independently extract data from the included studies. Data will be extracted using a standardised Excel (Microsoft, Albuquerque, USA) form, based on Cochrane recommendations for intervention reviews for RCTs and non-RCTs [21]. The extraction form will include information related to the reference, study methodology, participants, intervention, and outcomes. The extraction form will be piloted to ensure no data will be missing from the form. If data cannot be extracted from the original source material, the authors of the paper will be contacted to obtain the missing information.

### Risk of bias assessment

Two review authors will independently assess risk of bias for each individual study using either the Cochrane risk-of-bias tool for randomised trials (RoB 2.0 tool) for randomised trials [22, 23] or Risk Of Bias in Non-randomised Studies of Interventions (ROBINS-I) for non-randomised trials [24, 25]. The RoB 2.0 tool assesses the bias that can arise in randomised trials in five domains: randomisation process, deviations from intended interventions, missing outcome data, measurement of the outcome, and selection of the reported result [23]. The ROBINS-I tool assesses bias within 3 domains, namely pre-intervention, at intervention, and post-intervention [25]. Any discrepancies between the two reviewers will be assessed by discussion and if not agreed, the third reviewer (JS).

### Planned methods of analysis

#### Characteristics of included studies

Descriptive statistics and study population characteristics for all included studies will be produced and reported in a table; these will include article characteristics and clinical and methodological variables, such as average age, follow-up, and main trial outcome.

#### Meta-analysis

If sufficient studies for a specific drug group (antihypertensive, antiplatelet, antihyperlipidemic, and anticoagulant) are identified, we will conduct a meta-analysis for those groups. Meta-analyses will be conducted separately for randomised controlled trials and non-randomised studies of interventions. When a meta-analysis will be performed, we will use a random effects model to calculate the aggregate effect. If significant inconsistency is present in the direction of effect, a meta-analysis will not be performed.

#### Measures of treatment effect

Studies might utilise different methodologies to assess their outcome. To address this, we will do the following. For binary outcomes (e.g. hospitalisation) a pooled odds ratio will be calculated. For continuous outcomes that are determined through different methods (e.g. quality of life assessment), the standardised mean difference is used. For continuous outcomes that are assessed through the same test, we will use the mean difference. Data analysed using Poisson regression, count data, will be pooled similarly to other relative risk outcomes.

#### Assessment of heterogeneity

Clinical heterogeneity will be assessed and determined by two independent reviewers and statistical heterogeneity will be assessed by quantifying inconsistency I^2^ (Cochran Q test) [26].

#### Sensitivity analysis

If sufficient data are available, sensitivity analyses will be performed to ascertain how or if the results change under different assumptions, namely inclusion/exclusion of specific settings (nursing home or hospital), high risk of bias studies, and studies that included a younger population.

#### Subgroup analysis and meta-regression

If sufficient studies (more than 10 within a specific drug group) are available, we will conduct meta-regression analyses looking at the influence of various baseline characteristics, e.g. baseline medication burden and average medication reduction. If meta-regression is not feasible, we will report where possible the subgroup analysis of the following groups: sex, age (75–84 and 85 years and over), medication classes (e.g. beta blockers), cognitive functioning (no cognitive decline vs any functional cognitive decline), renal status (stage 1-2 vs stage 3 and over), health care provider delivering the intervention (doctor, nurse practitioner, pharmacist, other), and the frailty (no frailty vs frailty).

#### Publication and reporting bias

Reporting biases will be assessed by comparing published reports with protocols (either published in journals or obtained from databases) and the use of contour-enhanced funnel plots to identify small-study effects where appropriate [27].

#### Strength of evidence assessment

To assess the certainty of the body of evidence, we will use the GRADE approach (Grading of Recommendations Assessment, Development and Evaluation) as recommended by Cochrane [28].

#### Statistical software

All statistical analysis will be performed using R (meta and metafor packages) [29–31].

## Discussion

Despite recognition of the negative health impacts of problematic polypharmacy and increased interest in medication reduction to improve outcomes or reduce harm in older community-dwelling older patients, evidence is still scarce to support health care professionals in decision-making. Due to the nature of the intervention, potential negative outcomes, and the principle of *primum non nocere* (‘first, do no harm’), it is important to understand and summarise the short- and long-term health impacts of medication reduction. Furthermore, it is important to determine if there are any differential health outcomes in specific populations (e.g. sex, cognitive status, renal functioning) or which drugs are potentially safe to reduce [8]. The study described in this protocol seeks to summarise all currently available evidence that could help to address these questions and identify gaps in the evidence which might inform future research on this topic.

### Strengths and limitations of this approach

Although great care has been taken into developing the search strategy to identify all the relevant literature, it is known that deprescribing or medication reduction studies are hard to capture due to lack of consistent and universal search terms [16, 18]. Our search strategy uses a combination of title and abstract, keyword, and subheading search terms that are generally associated with medication withdrawal or reduction studies to increase the number of relevant records identified. In addition, screening of reference lists of included records will help us identify any studies not captured by the search strategy.

Due to the scarcity of randomised evidence on cardiovascular deprescribing interventions, this study will also assess data from observational studies. Non-randomised data are inherently subject to more bias compared to randomised study data. Patient or health care professional perceptions could influence the decision to initiate medication reduction, for example patients with underlying life-limiting pathologies might be more likely to discontinue medication than those whom are not resulting in a higher mortality rate in medication discontinuation groups if not or insufficiently controlled for. Different statistical approaches have been developed to adjust for these imbalances between groups using known confounders related to the indication, but this cannot obviously not be done for unknown confounders. However, data from well-designed observational studies can still provide good estimates of potential benefits and harms with minimal risk of bias [32].

Outcomes of interest for this study can be broadly grouped in safety and efficacy outcomes. Safety outcomes in medication reduction interventions are equivalent to efficacy outcomes of therapeutic interventions, e.g. major cardiovascular events. Efficacy outcomes in this study are those outcomes that are hypothesised to improve due to reducing the cardiovascular prescription exposure, which closely resemble safety outcomes in intervention trials, e.g. bleeding or falls. All-cause hospitalisation has been chosen as the primary outcome as it can be increased both in prescribing and medication reduction interventions, when not done correctly. Furthermore, hospitalisation is an impactful experience especially in the elderly, increasing the risk of developing new diseases (both somatic and psychological) and increases their dependency, negatively affecting autonomy and increases the risk of depression [33–35].

The focus of this study is medication reduction in older people, but health care setting could potentially influence outcomes due to potential differences in health care delivery. Our hypothesis is that the direction of effect and expected effect size should be similar across settings, due to randomisation or matching. A sensitivity analysis will be included to verify this hypothesis.

If enough studies are identified, we plan to undertake meta-regression analyses. Using the average of patient characteristics as covariates in such an analysis can result in ‘ecological bias’ and therefore should be interpreted with caution and not used to draw conclusions about the causal effects of specific patients’ characteristics. However, outcomes generated by meta-regression can be hypothesis generating and can be used to inform development of future studies [36].

Any amendments made to the protocol when conducting the review will be outlined in PROSPERO and reported in the final manuscript.

In summary, this study will provide a thorough review of all studies assessing the benefits and/or harms of cardiovascular medication reduction in older people. The results will be important for guideline developers and clinicians when making decisions on which medication prescription to withdraw and whom to consider for medication reduction interventions. Compared to results from the existing systematic reviews [15–18], this overarching summary of all relevant work will enhance decision-making as it will include a broader selection of cardiovascular drugs and outcomes to better reflect the complexity of the intervention in routine clinical practice. Results from the systematic review will be disseminated through conference abstracts, social media, and publication in a peer-reviewed journal, and a layman summary will be used for future public and patient involvement and will be made freely accessible on the website of the Nuffield Department of Primary Health Care Sciences. Findings will be used to inform researchers on potential gaps in the evidence which need to be addressed.

## Supplementary Information

**Additional file 1.**

**Additional file 2.**

## Data Availability

Not applicable

## Abbreviations

NRSI: Non-randomised studies of interventions
RCT: Randomised controlled trial
RoB 2.0: Risk of Bias Tool for randomised trials
ROBINS-I: Risk of Bias in Non-randomised Studies of Interventions
