# Supplementary material for: The health impacts of preventive cardiovascular medication reduction on older populations: protocol for a systematic review and meta-analysis
Source: Syst Rev. 2021 Jun 24;10:185. doi: 10.1186/s13643-021-01741-2 (PMC8229162; doi:10.1186/s13643-021-01741-2)
Supplement: Supplementary file 2 — Additional file 2. [file 13643_2021_1741_MOESM2_ESM.docx]

1 Clinical Trial/ or Randomized Controlled Trial/ or Controlled clinical trial/ or Multicenter study/ or Phase 3

clinical trial/ or Phase 4 clinical trial/ or exp RANDOMIZATION/ or Single Blind Procedure/ or Double Blind Procedure/

or Crossover Procedure/ or PLACEBO/ or Prospective Study/ (2155321)

2 (randomi?ed controlled trial* or rct or single blind* or double blind* or placebo*).tw. (601198)

3 (random* adj2 allocat*).tw. (43104)

4 ((treble or triple) adj blind$).tw. (1164)

5 1 or 2 or 3 or 4 (2322441)

6 Case Study/ or abstract report/ or letter/ (1214578)

7 case report.tw. (413531)

8 (Conference proceeding or conference abstract or editorial or letter or note).pt. (6377227)

9 6 or 7 or 8 (6824551)

10 5 not 9 (1722495)

11 Clinical study/ or case control study/ or Longitudinal study/ or Retrospective study/ or Cohort analysis/

(1744297)

12 (Cohort adj (study or studies)).mp. (304476)

13 (Case control adj (study or studies)).tw. (133245)

14 (follow up adj (study or studies)).tw. (63118)

15 (observational adj (study or studies)).tw. (166172)

16 (epidemiologic$ adj (study or studies)).tw. (105922)

17 (cross sectional adj (study or studies)).tw. (216800)

18 11 or 12 or 13 or 14 or 15 or 16 or 17 (2291161)

19 (cohort stud* or cohort analys* or prospective or retrospective or follow up stud* or case control or

case-control or longitudal).tw. (1945943)

20 10 or 18 or 19 (4327521)

21 exp *aged/ or exp *geriatrics/ or exp *geriatric nursing/ or (centarian* or centenarian* or elder* or eldest or

frail* or geriatri* or nonagenarian* or octagenarian* or octogenarian* or old age* or older adult* or older age* or

older female* or older male* or older man or older men or older patient* or older people or older person* or older

population or older subject* or older woman or older women or oldest old* or senior* or senium or septuagenarian* or

supercentenarian* or very old*).ti,ab,kw. (757248)

22 exp antihypertensive agent/ or exp hydroxymethylglutaryl coenzyme A reductase inhibitor/ or exp thrombocyte

aggregation/ or exp anticoagulant agent/ (1388367)

23 (diuretic* or statin* or antiplatelet* or antithrombotic* or cardiovascular medication* or oral anticoagulant* or

warfarin or beta-blocker* or ACE inhibitor* or diuretic or rivaroxaban or dabigatran or lipid modifying drugs or aspirin

or clopidogrel or prasugrel or ticagrelor or cangrelor or edoxaban or apixaban or ARB or angiotensin or

antihypertensive* or anticoagulant*).ti,ab. (549448)

24 22 or 23 (1547459)

25 21 and 24 (52829)

26 exp inappropriate prescribing/ or exp deprescription/ or exp treatment withdrawal/ or exp drug dose/ (785018)

27 (medication reduc* or medication with* or medication discontin* or deprescrib* or stopping treatment or

discontin* or withdraw*).ti,ab. (376698)

28 26 or 27 (1067604)

29 20 and 25 and 28 (3929)
